# Feasibility of using stereotactic body radiation therapy for unresectable soft tissue tumors of the trunk

**DOI:** 10.18632/oncotarget.25539

**Published:** 2018-06-12

**Authors:** Eun Kyung Paik, Mi-Sook Kim, Chul-Koo Cho, Hyung Jun Yoo, Won Il Jang, Young-Seok Seo, Sung-Ho Jin, Dae Geun Jeon, Dong Han Lee

**Affiliations:** ^1^ Department of Radiation Oncology, Korea Institute of Radiological and Medical Sciences, Seoul, Korea; ^2^ Department of Radiation Oncology, Seoul National University Hospital, Seoul, Korea; ^3^ Department of Surgery, Korea Institute of Radiological and Medical Sciences, Seoul, Korea; ^4^ Department of Orthopedic Surgery, Korea Institute of Radiological and Medical Sciences, Seoul, Korea; ^5^ CyberKnife Center, Korea Institute of Radiological and Medical Sciences, Seoul, Korea

**Keywords:** soft tissue tumor, sarcoma, stereotactic body radiation therapy, unresectable, trunk

## Abstract

**Purpose:**

To evaluate the feasibility of stereotactic body radiation therapy (SBRT) for unresectable soft tissue tumors of the trunk.

**Materials and Methods:**

Between January 2002 and December 2008, 23 patients with 36 lesions of soft tissue tumors, which were located in the trunk and not suitable for resection, underwent SBRT. Among the 36 lesions, 31 were malignant and 5 were benign. The median tumor volume was 24 cm^3^ (range, 2.6–213 cm_3_). SBRT doses ranged from 20 to 48 Gy in 1–5 fractions.

**Results:**

With a median follow-up of 73 months, the overall survival (OS) and local control (LC) rates at 5 years were 39% and 52%, respectively. For malignant tumors, the OS and LC rates at 5 years were 28% and 47%, respectively. For benign tumors, the OS and LC rates at 5 years were 80% and 100%, respectively. There was no acute toxicity of grade ≥3. One case of grade 3 late skin toxicity was reported 10 months after SBRT.

**Conclusion:**

SBRT may be an effective and safe treatment modality for the local control of unresectable soft tissue tumors of the trunk including tumors of a benign nature.

## INTRODUCTION

Soft tissue sarcomas (STSs) are rare tumors arising from connective tissues that can occur in any anatomical site. They represent about 1% of cancers diagnosed in the US with an annual incidence of about 12,000 cases [1]. The extremities are the most common sites of presentation, accounting for approximately 60% of cases, followed by trunk (15–20%), retroperitoneum (10–15%), and the head and neck (8%) [2]. The aim of the treatment of STS is to achieve complete eradication of the tumor with optimal function preservation and minimal treatment-related toxicities. Several randomized trials have been performed and have established conservation surgery combined with radiation therapy (RT) as the standard management for most STSs of the extremities and trunk [3–5]. However, the cumulative probability of local recurrence at 5 years in extremity STS, as reported in large series, remains as high as 20% [3–6].

Due to their deep location, presentation of soft tissue tumors in the trunk is often delayed until the tumor becomes very large. Analysis of the Surveillance Epidemiology and End Results (SEER) database showed that the median tumor size in the case of retroperitoneal tumors is 15.5 cm (range, 0.5–99.5 cm) [7]. Therefore, it is often difficult to achieve complete resection with an optimal margin. As a result, following frequent local recurrences and repeated operations, the tumor eventually becomes inoperable. For inoperable STS, palliative chemotherapy has been the long-standing treatment option. In certain cases, such as for well-localized tumors in the extremities, the overall survival (OS) rate at 2 years with palliative chemotherapy is reported to be up to 28–31% [8]. Despite this, only a few patients achieve an objective response. In situations where the effect of chemotherapy for STS is uncertain, RT is the choice for local treatment. However, for the same reason of difficulty with surgery, sufficient doses of radiation may not be administered for STSs of the trunk, resulting in radiation treatment administered mostly with a palliative aim, such as pain relief.

Owing to remarkable advances in technology for tumor imaging and radiation delivery systems, it has become possible in recent years to treat a variety of cancers with stereotactic body radiation therapy (SBRT) with high-dose per fraction. One of the advantages of treating tumors with SBRT over the conventional external beam radiation therapy (EBRT) is that SBRT precisely irradiate tumors while allowing tight margin of surrounding normal tissues. Furthermore, because the α/β ratio of STS is known to be relatively low with a range of 1.4–5.4 [9], the therapeutic gain with SBRT in 1–5 fractions may be greater than that with the conventional multi-fractionated radiotherapy. Despite these theoretical advantages, there has been little definitive clinical investigation. The purpose of this study was to evaluate the feasibility and safety of SBRT for unresectable soft tissue tumors of the trunk, including malignant tumors and pathologically benign tumors exhibiting malignant behavior.

## RESULTS

### Treatment outcomes

**Figure 1:**
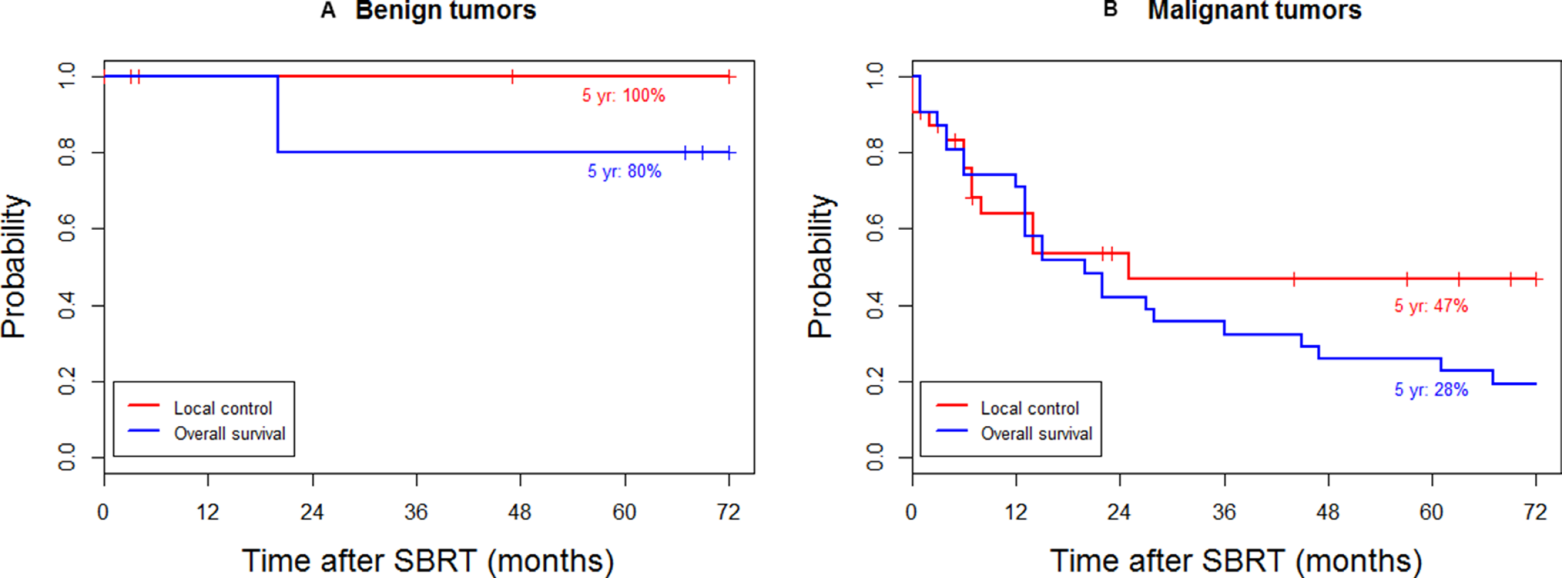
Local control and overall survival for (**A**) benign and (**B**) malignant tumors.

The treatment outcomes of SBRT to 23 patients with 36 lesions of unresectable soft tissue tumors located in the trunk were analyzed. The baseline patient and tumor characteristics are shown in Table 1. With a median follow-up of 73 months for patients alive at the last follow-up, the local control (LC) rate at 5 years was 52%, and the OS rate at 5 years was 39%. For malignant tumors (18 patients with 31 lesions), the OS and LC rates at 5 years were 28% and 47%, respectively (Figure 1A). For benign tumors (5 patients with 5 lesions), the OS and LC rates at 5 years were 100% and 80%, respectively (Figure 1B).

**Table 1:** Baseline patient and tumor characteristics

Characteristics	Range (median)	No. of patients (%) (*N* = 23)	No. of lesions (%) (*N* = 36)
**Gender**			
Male		14 (61)	
Female		9 (39)	
**Age**	23–72 years (41)		
**Location**			
Chest			15 (42)
Abdomen			8 (22)
Pelvis			13 (36)
**Presentation**			
Primary		3 (13)	3 (8)
Recurrent		20 (87)	33 (92)
**Tumor characteristics**			
Benign		5 (22)	5 (14)
Malignant		18 (78)	31 (86)
**Histology**			
Undifferentiated pleomorphic sarcoma		5 (22)	12 (33)
Synovial sarcoma		4 (17)	7 (19)
Epithelioid sarcoma		2 (9)	3 (8)
Hemangiopericytoma		1 (4)	3 (8)
Others		11 (48)	11 (29)
**Tumor volume**	2.6–213 cm^3^ (24)		

In order to investigate the effect of prognostic factors on LC and OS rates, univariate analysis was performed. Only tumor characteristics of a benign or malignant nature had a significant impact on OS (*P* = 0.033), and no significant prognostic factor was associated with LC.

The 5 benign tumors were treated with SBRT because they were unresectable due to their location and previous operation histories (Table 2). Patient 1 had undergone 7 previous surgeries for different lesions, and the treated tumor in the buttock area recurred 8 months after surgery. The size of the mass was increasing and the patient was complaining symptoms of pain. Patient 2 had a recurrent tumor in the paraspinal (sacral) area 1 year after surgery. The tumor showed a rapid growing pattern, and the patient was referred for RT hoping to slow down the growth or to reduce the tumor volume. Patient 3 had undergone 4 previous surgeries for different lesions, and the treated tumor in the chest wall recurred 9 years after surgery. The patient refused surgery. Patient 4 had a tumor in the paraspinal (lumbar) area, which was followed up for 15 years before radiating pain developed, and was treated with SBRT. Patient 5 had a tumor in the paraspinal (lumbar) area. Although it was asymptomatic, the size of the tumor continuously increased during 3 years of follow-up. One patient (patient 5) died during follow-up 20 months after SBRT due to hepatocellular carcinoma. All of the lesions treated with SBRT remained stable, symptoms were relieved, and none of the lesions needed further treatment. Except for the patient who died 20 months after SBRT, long-term LC of 73–128 months was achieved.

**Table 2:** Characteristics of benign soft tissue tumors

Patient No.	Sex/Age	Histology	Location	Volume (cm^3^)	Dose (Gy/fx)	BED (Gy_3_)	F/U^a)^ (M)
1	F/50	Fibromatosis	Buttock	38.4	33/3	92	128
2	M/32	Giant cell tumor	Paraspinal	143.8	42/3	143	102
3	M/32	Neurofibroma	Chest wall	9.5	24/3	53	86
4	M/37	Hemangioblastoma	Paraspinal	11.2	24/3	53	73
5	M/56	Neurogenic tumor	Paraspinal	33.7	27/3	65	20^b)^

Abbreviations: BED, biologically equivalent dose; F/U, follow-up after SBRT; M, months

a)All patients achieved local control during follow-up.

b)Patient 5 died from hepatocellular carcinoma at 20 months after SBRT.

Five re-irradiated malignant cases were also included in this study (Table 3). Four patients had undergone previous operations. Among them 1 patient received preoperative RT and 3 patients received postoperative RT. One patient without previous surgery had received previous SBRT of 20 Gy in 1 fraction. All 5 patients showed local recurrences and distant metastases during follow-up. Local recurrence occurred in all lesions within 2 years, and all patients expired within 3 years. The median LC for the re-irradiated lesions was 14 months (range, 4–23 months). The median OS was 22 months (range, 12–28 months).

**Table 3:** Characteristics of re-irradiated cases

Sex/Age	Histology	Location	Volume (cm^3^)	Previous RT dose (Gy/fx)	SBRT dose (Gy/fx)	Duration^a)^ (M)	OS^b)^ (M)	LC^c)^ (M)	Toxicity y ≥ Gr 3
F/31	Synovial sarcoma	Pelvis	33.7	30/10	24/3	1	28	14	None
F/52	Malignant schwannoma	Back	213.8	60/30	30/3	64	27	23	Skin^d)^
M/63	Undifferentiated pleomorphic sarcoma	Pelvis	9.4	60/30	40/5	25	22	14	None
F/34	Myofibroblastic sarcoma	Buttock	124.6	45/15	39/3	19	20	4	None
M/31	Synovial sarcoma	Paraspinal	59.3	20/1	30/3	20	12	7	None

Abbreviations: RT, radiation therapy; SBRT, stereotactic body radiation therapy; OS, overall survival; M, months; LC, local control; Gr, grade.

a)Duration; duration between previous RT and re-irradiation.

b)All patients expired within 3 years after SBRT.

c)All lesions recurred within 2 years after SBRT.

d)Grade 3 skin toxicity occurred at 10 M after SBRT.

### Toxicity

Toxicities were investigated and graded according to the Common Terminology Criteria for Adverse Events (CTCAE) v4.0. There were no severe acute gastrointestinal, genitourinary, or skin toxicities of grade ≥3. Grade 3 late skin toxicity was observed in 1 re-irradiated case 10 months after SBRT treatment (Table 3). The tumor was treated with SBRT with a dose of 30 Gy in 3 fractions delivered in 3 consecutive days. The patient had undergone several previous surgeries due to repeated recurrences, and was previously treated with 60 Gy in 30 fractions of postoperative RT 64 months before the retreatment with SBRT. The patient experienced wet desquamation of the skin from 1 month after SBRT. Ten months after the SBRT treatment, the patient was hospitalized for antibiotic treatment due to radiation necrosis and infections of the skin in the treated area. No radiation-induced secondary malignancies were observed.

## DISCUSSION

In the present study, the SBRT of unresectable STS of the trunk with prescribed doses of 20–48 Gy in 1–5 fractions resulted in an LC rate of 52% and an OS rate of 39% at 5 years. The specific dose prescriptions and biologically equivalent doses (BEDs) for malignant tumors initially treated with SBRT are shown in Table 4. Due to the location of the tumor and nearby organs at risk, various dose fractionations were applied. When it is assumed that the α/β ratio of STS is 4 [10, 11], the total dose converted to equivalent doses in 2 Gy-fractions (EQD2) was 72–240 Gy_4_ (median 144 Gy_4_). For cases of R1 resection margins (microscopic positive margins), R2 resection margins (macroscopic positive margins), and unresectable tumors treated with conventional RT, 5-year LC rates are 39–52% [12–14]. In a retrospective study by Youssef *et al.* [12], tumors in the retroperitoneum and deep trunk were treated with surgery plus conventional EBRT, with or without brachytherapy. RT doses were EBRT 52.2 Gy or EBRT 42 Gy plus brachytherapy 16 Gy, and showed a 5-year OS rate of 48% and a 5-year LC rate of 52%. In a study by Kepka *et al.* [13], tumors in the extremities, retroperitoneum, head and neck, and the truncal wall were treated with conventional EBRT, with or without chemotherapy. With a median RT dose of 64 Gy (range, 25–87.5 Gy), they showed a 5-year OS rate of 35% and a 5-year LC rate of 45%. In a study by Feng *et al.* [14], tumors in the retroperitoneum, pelvis, and deep trunk were treated with EBRT, with or without surgery. The median RT dose was 56.4 Gy (range, 7–73 Gy), and the outcome was a 5-year OS rate of 12% and a 5-year LC rate of 39%. The results of SBRT for sarcomas of spine have been reported [15–17], but to our knowledge, our present study is the first one to investigate the SBRT for the sarcomas of the trunk. The overall results of our study were comparable to the above mentioned previously reported results of conventional fractionated radiotherapy [12–14]. As shown in Table 4, the BED values calculate using α/β = 4 and 10 varied considerably. However, considering the high dose when converted to BED with α/β = 4, an LC of approximately 50% seems low. Initially, when we decided on the SBRT dose fractionations, we converted the doses using α/β = 10. As a result, the SBRT doses we used are slightly higher than the conventional doses used in the literature. Therefore α/β may not be as low as 4, as suggested from the literature. However, in light of the increasing evidence that the linear-quadratic model is not valid for irradiation with a large dose/fraction, there are limitations in interpreting the results using BEDs.

**Table 4:** Details of SBRT doses of initially treated malignant STSs

Fractions	Total dose (Gy)	No. of patients treated	BED (Gy_4_)	BED (Gy_10_)
1	20 24 26	3 1 3	120 168 195	60 82 94
2	26 28 32	1 1 2	111 126 160	60 67 83
3	27 33 36 39 45 48	2 1 4 5 2 1	88 124 144 166 214 240	51 69 79 90 113 125

Abbreviations: SBRT, stereotactic body radiation therapy; STS, soft tissue sarcoma; BED, biologically equivalent dose.

There were 5 cases of benign soft tissue tumors included in our study. Although pathologically benign, some of the tumors behaved like malignant tumors from the point of view of local invasiveness or multiple recurrences after surgery. Benign tumors fall under the radiobiological category of late-responding tissues, and are assumed to have a low α/β ratio [18]. Therefore, hypofractionated radiotherapy may be more effective than conventional fractionated radiotherapy for benign tumors. The benign soft tissue tumors of 5 patients were unresectable owing to the location of the tumors and previous operation histories. Our study showed excellent LC and OS rates for benign tumors treated with SBRT with a dose of 24–42 Gy in 3 fractions delivered on consecutive days. If converted into EQD2 with α/β = 3, the doses were 53–143 Gy_3_. All of the treated lesions remained stable after treatment with SBRT, and none of the lesions needed further treatment. One patient died during follow-up due to hepatocellular carcinoma, but the treated lesion was stable 20 months after SBRT. None of the patients experienced severe complications of grade ≥3. Therefore, SBRT might be a promising treatment modality for unresectable benign soft tissue tumors in patients with a history of several operations and those presenting with uncontrolled pain. However, the number of patients in our study is small, and further evidence needs to be accumulated.

In our study, 5 patients were re-irradiated for recurrence with SBRT. Most of the patients had undergone several previous surgeries due to multiple recurrences. Studies in which patients were retreated with EBRT show serious complication rates of 42–60%. Since SBRT allows delivery of high doses of radiation to limited volumes of tissue whilst sparing the surrounding normal tissues, SBRT may be applied for re-irradiation. In similar rationale, brachytherapy has been used for recurrent STS cases in the extremities after previous radiation treatment. However, there may be some limitations for applying brachytherapy due to large tumor volumes, restrictions in catheter placement due to normal tissue anatomy, and risk of radiation injury to normal tissues in direct contact with the catheters [19]. Previous studies of brachytherapy for re-irradiation shows results derived from diverse tumor locations and tumor sizes. Therefore, direct comparison of our results with the results from other studies would be difficult. However, it would be useful to look into these studies for some insights regarding complications. In a study of brachytherapy from Memorial Sloan-Kettering Cancer Center, the 5-year OS and LC rates were 85% and 68%, respectively [5]. However, a 12.5% rate of serious complications was observed with a median follow-up of 3 years. A study from the MD Anderson Cancer Center suggested that re-irradiation does not clearly improve outcome after surgical excision alone, but in fact increases complications [20]. Using brachytherapy, patients were treated with 45–50 Gy. The 5-year LC and 5-year disease-specific survival rates were 51% and 65%, respectively, but serious complication rates were as high as 75%. In a study from the University of Florida, patients were retreated with radiation, including brachytherapy, for recurrent STSs [21]. The median RT dose was 50.4 Gy (range, 38.0–66.0) at presentation, and 57.6 Gy (43.2–66.0) at recurrence. For retreatment, the 5-year OS and LC rates were 40% and 18%, respectively, but there was a 50% incidence of serious complications. In our study, 1 patient showed grade 3 skin toxicity 10 months after SBRT. The patient was previously treated with 60 Gy in 30 fractions 64 months before being retreated with SBRT of 30 Gy in 3 fractions. However, the patient had also undergone several previous surgeries on the treated site, and the toxicity may have resulted from combined treatments. Therefore, even when it is possible to treat with a palliative aim, care must be taken not to cause complications.

In conclusion, the present study demonstrated that SBRT is an effective and safe treatment modality for unresectable soft tissue tumors of the trunk, with a low risk of severe toxicity. SBRT may have role in the management of benign soft tissue tumors, showing malignant behavior by providing satisfactory progression-free survival without the need for further treatment. However, for re-irradiation cases, more evidence needs to be accumulated before drawing any conclusions.

## MATERIALS AND METHODS

### Patients

Twenty-three patients with unresectable soft tissue tumors located in the trunk were treated with SBRT using CyberKnife (Accuray Inc., Sunnyvale, CA, USA) during January 2002 to December 2008 at our institute. The medical records of a total of 23 patients with 36 lesions located in the trunk that were unsuitable for resection were retrospectively reviewed. Patients who were treated with conventional EBRT followed by SBRT boost were excluded. This study was approved by the Institutional Review Board of our institute.

Fourteen patients were male and 9 were female. Ages ranged from 23 to 72 years with a median of 41 years. The size of the tumor ranged from 2.6 to 213 cm^3^, with a median of 24 cm^3^. The locations of the lesions by anatomical site were diverse and were as follows. Fifteen lesions were in the chest, including 4 lung lesions, 3 chest wall lesions, 3 axilla lesions, 3 paraspinal lesions, 1 back lesion, and 1 paracardiac lesion. Eight lesions were in the abdomen, including 5 paraspinal lesions, 1 paraaortic lesion, 1 anterior abdominal wall lesion, and 1 liver lesion. Thirteen lesions were in the pelvis, including 4 intrapelvic lesions, 3 buttock lesions, 2 parasacral lesions, 2 inguinal lesions, 1 perineal lesion, and 1 perianal lesion. Three were primary cases and 33 were recurrent cases. Five re-irradiated cases were also included. Five had benign tumor characteristics and 31 were malignant. The histology of the tumors was heterogeneous, including 12 undifferentiated pleomorphic sarcoma, 7 synovial sarcomas, 3 epithelioid sarcomas, 3 hemangiopericytomas, 1 liposarcoma, 1 giant cell tumor, 1 fibromatosis, 1 neurofibroma, 1 hemangioblastoma, 1 malignant schwannoma, 1 rhabdomyosarcoma, 1 follicular dendritic cell sarcoma, 1 myofibroblastic sarcoma, 1 neurogenic tumor, and 1 malignant peripheral nerve sheath tumor.

### Stereotactic body radiation therapy

Patients were treated with SBRT using the CyberKnife system. The gross tumor identified on the simulation computed tomography (CT) scan was defined as the gross tumor volume (GTV). A margin of 0–4 mm was added to the GTV for the planning target volume (PTV). SBRT doses were prescribed at an isodose line (64–83% of the maximum dose) that covered at least 97% of the PTV. A total dose of 20–48 Gy (median 32 Gy) in 1–5 fractions was prescribed. The overall treatment time for those except single treatment cases were 2–11 days. Most of the patients received treatment on consecutive days excluding weekends. For the patients who did not receive consecutive treatments (4 patients, 5 lesions), 1 patient received 2 fractions of treatment 11 days apart due to nausea and vomiting after the first treatment, another patient had 2 lesions treated with 3 fractions each alternatively for a total of 7 days, and the other 2 patients received 3 fractions of treatment each with at least 48 hours apart. The BED, when calculated with α/β = 10, ranged from 43 Gy_10_ to 125 Gy_10_, with a median BED of 79 Gy_10_, and with α/β = 4, BED ranged from 72 Gy_4_ to 240 Gy_4_, with a median BED of 144 Gy_4_. If converted into EQD2, the prescribed dose was 36–104 Gy_10_ with a median EQD2 of 66 Gy_10_ for α/β = 10, and for α/β = 4, the prescribed dose was 48–160 Gy_4_, with a median EQD2 of 96 Gy_4_.

### Statistical analysis

LC and OS rates for all patients were calculated from the date of the SBRT using the Kaplan-Meier method. Univariate analysis was performed using the log-rank test to identify significant prognostic factors for LC and OS. For all analyses, two-sided tests of significance were used with *P* values < 0.05 considered significant. All statistical analyses were performed using the Statistical Package for the Social Sciences (version 14.0; SPSS, Inc. Chicago, IL, USA).

## Abbreviations

STS: soft tissue sarcoma
RT: radiation therapy
OS: overall survival
SBRT: stereotactic body radiation therapy
EBRT: external beam radiation therapy
LC: local control
BED: biologically equivalent dose
EQD2: equivalent doses in 2 Gy-fractions
GTV: gross tumor volume
PTV: planning target volume
